# Prenatal Genome-Wide Cell-Free DNA Screening: Three Years of Clinical Experience in a Hospital Prenatal Diagnostic Unit in Spain

**DOI:** 10.3390/genes15050568

**Published:** 2024-04-28

**Authors:** Laia Pedrola Vidal, Mónica Roselló Piera, Carla Martín-Grau, Juan S. Rubio Moll, Rosa Gómez Portero, Beatriz Marcos Puig, Jose V. Cervera Zamora, Ramiro Quiroga, Carmen Orellana Alonso

**Affiliations:** 1 Genetics Unit, Hospital Universitari i Politècnic La Fe, 46026 Valencia, Spain; pedrola_lai@gva.es (L.P.V.); cervera_jos@gva.es (J.V.C.Z.); orellana_car@gva.es (C.O.A.); 2 Translational Genetics Research Group, Instituto de Investigación Sanitaria La Fe (IISLAFE), 46026 Valencia, Spain; carla_martin@iislafe.es; 3 Obstetrics and Gynecology Unit, Hospital Universitari i Politècnic La Fe, 46026 Valencia, Spain; rubio_jua@gva.es (J.S.R.M.); gomez_rospor@gva.es (R.G.P.); marcos_bea@gva.es (B.M.P.); quiroga_ram@gva.es (R.Q.)

**Keywords:** prenatal cfDNA screening, genome-wide, rare autosomal aneuploidies, copy-number variations, trisomy

## Abstract

Genome-wide prenatal cell-free DNA (cfDNA) screening can be used to screen for a wide range of fetal chromosomal anomalies in pregnant patients. In this study, we describe our clinical experience with a genome-wide cfDNA assay in screening for common trisomies, sex chromosomal aneuploidies (SCAs), rare autosomal aneuploidies (RAAs), and copy-number variations (CNVs) in about 6000 patients over a three-year period at our hospital’s Prenatal Diagnostic Unit in Spain. Overall, 204 (3.3%) patients had a high-risk call, which included 76 trisomy 21, 21 trisomy 18, 7 trisomy 13, 29 SCAs, 31 RAAs, 31 CNVs, and 9 cases with multiple anomalies. The diagnostic outcomes were obtained for the high-risk cases when available, allowing for the calculation of positive predictive values (PPVs). Calculated PPVs were 95.9% for trisomy 21, 77.8% for trisomy 18, 66.7% for trisomy 13, 10.7% for RAAs, and 10.7% for CNVs. Pregnancy and birth outcomes were also collected for the majority of RAA and CNV cases. Adverse perinatal outcomes for some of these cases included preeclampsia, fetal growth restriction, preterm birth, reduced birth weight, and major congenital structural abnormalities. In conclusion, our study showed strong performance for genome-wide cfDNA screening in a large cohort of pregnancy patients in Spain.

## 1. Introduction

The clinical introduction of prenatal cell-free DNA (cfDNA) screening, also known as noninvasive prenatal testing/screening (NIPT/NIPS), over a decade ago led to a global transformation in prenatal screening for pregnant patients. Studies have consistently shown improved performance with prenatal cfDNA screening over traditional screening approaches for common trisomies (trisomies 21, 18, and 13). Many professional medical societies recommend cfDNA screening as a prenatal screening approach for common trisomies for all pregnant patients [1,2,3,4,5,6]. The American College of Medical Genetics and Genomics strongly recommends prenatal cfDNA screening for common fetal trisomies over traditional screening methods for all patients with singleton and twin gestations and for fetal sex chromosome aneuploidies (SCAs) in singleton pregnancies [3]. 

Previous studies reported that the screening focused on common trisomies and SCAs could not detect about 17% of other clinically relevant chromosomopathies [7]. In recent years, the scope of prenatal cfDNA screening has expanded to include optional screening for rare autosomal aneuploidies (RAAs) and copy-number variations (CNVs). Recent publications have reported their clinical experience with screening for these rarer chromosomal abnormalities [8,9,10,11,12,13,14,15], and some of them observed that these additional findings were related to perinatal outcomes. Harasim et al. found that half of their RAA cases had signs of either placental insufficiency or intrauterine death [8]. They also found that prenatal cfDNA screening for CNVs enabled the detection of unbalanced translocations and relevant maternal health conditions. Mossfield et al. showed that many of their patients had adverse pregnancy outcomes, including fetal growth restriction (FGR), intrauterine fetal demise, and preterm birth, when RAAs were detected [9]. Moreover, the TRIDENT study in the Netherlands found that most of these additional findings had a clinical impact [15]. The majority of fetal chromosomal aberrations were pathogenic and associated with severe clinical phenotypes, and over half of the confined/assumed confined placental mosaicism (CPM) cases were associated with adverse perinatal outcomes [15].

In this retrospective cohort study, we describe our clinical experience with prenatal genome-wide cfDNA screening at La Fe University and Polytechnic Hospital (Valencia, Spain) over a three-year period. We detail the performance of a paired-end sequencing-based cfDNA screening assay in the detection of genome-wide fetal anomalies and discuss pregnancy and birth outcomes for patients who received a high-risk result.

## 2. Materials and Methods

**Figure 1:**
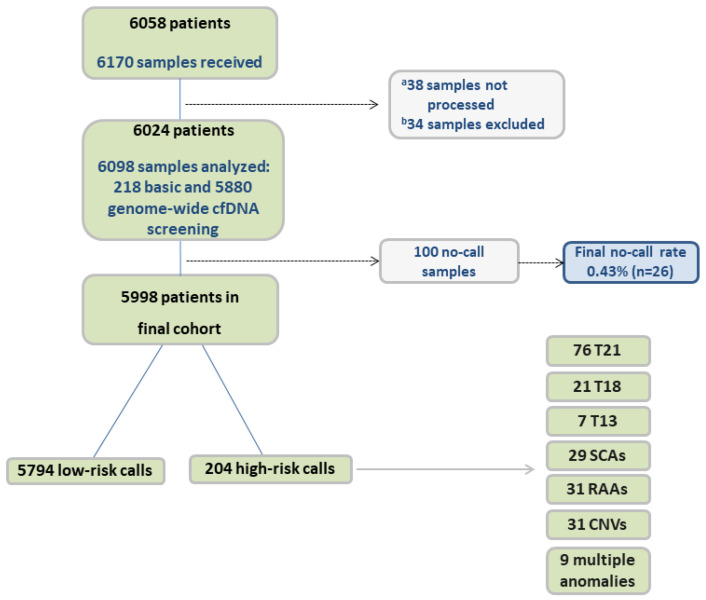
Study flowchart. cf, cell-free; T, trisomy; SCA, sex chromosome aneuploidy; RAA, rare autosomal aneuploidy; CNV, copy-number variant. ^a^ Samples were not processed due to several factors, including samples in a wrong tube (broken or unidentified) and sample clotting. ^b^ Samples from patients with exclusion criteria.

Samples were obtained from pregnant patients at 21 public maternity health centers throughout the community of Valencia (Alicante, Castellón, and Valencia) from 1 March 2020 to 31 December 2022. The study flowchart is outlined in Figure 1. Samples from singleton, twin, and vanishing twin (VT) pregnancies were included. In 2020, prenatal cfDNA screening was offered to patients with a risk of 1/50–1/270 following combined first trimester screening (cFTS). In 2021, this was expanded to 1/1000 following cFTS. It was also offered to patients with a high risk in the second-trimester screening (cSTS) or late pregnancies without cFTS performed, patients with a previous pregnancy with aneuploidies on chromosomes 21, 18, or 13, an X-linked disease, and parental translocation carriers. Some other patients were included following obstetric criteria, such as triple gestations, advanced maternal age, vanishing twins, miscarriage, placenta dysfunction, ultrasound anomalies, and pregnant women who rejected an invasive technique. Other patients were included with unknown/not specified medical prescription. Eligible patients were older than 18 years and ≥10 weeks of gestation. The study was conducted in accordance with the Declaration of Helsinki, and informed consent was obtained from all participating individuals.

For the cfDNA screening analysis, frozen plasma samples were processed using the VeriSeq™ NIPT Solution v2 assay (Illumina, Inc., San Diego, CA, USA) [16]. Briefly, cfDNA was extracted from samples and underwent library preparation. Library products were then quantified before being pooled and sequenced. Following this, sequencing data was analyzed using VeriSeq NIPT Assay Software v2 and a report was generated [17]. The assay provides an Anomaly Detected (high-risk) or No Anomaly Detected (low-risk) result for common trisomies, RAAs, and CNVs, with an option to request the reporting of SCAs. Patients consented to either basic screening (common trisomies) or genome-wide screening (inclusion of RAAs and CNVs). A fetal fraction (FF) estimate was provided during sample analysis, along with log likelihood ratio (LLR) score. LLRs are used to classify samples as Anomaly Detected or No Anomaly Detected based on a predetermined region-specific threshold [10]. Mosaic ratio is the proportion of the aneuploid fetal DNA and was provided as part of the analysis with the VeriSeq NIPT Solution v2 analysis software [17].

Clinical follow-up was attempted for all high-risk cases. Clinical outcome data included genetic results from chorionic villus sampling (CVS), amniotic fluid, products of conception (POC), and postnatal testing of tissues. Confirmatory methodologies used included fluorescent in situ hybridization (FISH), karyotyping, array, and quantitative fluorescent PCR (QF-PCR). We obtained information on pregnancy outcomes, including complications such as preeclampsia and FGR, timing of delivery or spontaneous abortion, and adverse perinatal outcomes such as low birth weight or admission to the neonatal intensive care unit (NICU). Although follow-up was not carried out for the low-risk cases, it is probable that we would have been informed of any false-negative results.

Statistical analysis was carried out using the statistical software package SPSS version 25.0 for Windows (SPSS, Chicago, IL, USA). Furthermore, 95% confidence intervals (Cis) were calculated along with a theoretical positive predictive value (PPV) range using the assumption that all unknown outcomes were either true positives (upper PPV) or false positives (lower PPV).

## 3. Results

A total of 6098 samples were analyzed during the study period, 218 (3.6%) of which had basic cfDNA screening and 5880 (96.4%) had genome-wide cfDNA screening. In 88 cases, the result obtained with the first sample was inconclusive (no-call result). A second sample was obtained and analyzed in 74 of these cases, of which 12 returned a no-call result and 62 cases obtained a result; 4 of these had a high-risk cfDNA screening call (1 del (7), 2 trisomy 16, and 1 trisomy 21). Overall, 100 (1.6%) samples had a no-call result. In terms of patients/pregnant women, our study had a final no-call rate of 0.43% (n = 26) and a final study cohort of 5998 patients (Figure 1). FFs ranged from 2 to 34%, with an average of 10.09% (± 4.32%). Demographics for these 5998 patients are shown in Table 1, with test indications shown in Table 2. Regarding the type of pregnancy, 5851 (97.55%) were singleton pregnancies, 121 (2.02%) were twin pregnancies, and 26 (0.43%) were VT pregnancies.

According to the results, there were 5794 (96.6%) low-risk results and 204 (3.4%) high-risk calls, which included 76 trisomy 21, 21 trisomy 18, 7 trisomy 13, 29 SCAs (15 X0, 6 XXX, 4 XXY, 4 XYY), 31 RAAs, 31 CNVs, and 9 cases with multiple anomalies. Details of results for the common trisomies and SCAs are shown in Table 3. 

The screen-positive rate (SPR) for trisomy 21 was 1.27% (76/5998). Of these 76 cases, 74 were singleton and 2 were twin pregnancies. FFs for trisomy 21 cases ranged from 2 to 19%. Overall, 70 cases were confirmed to be true positives (TPs) and 3 were false positives (FPs), giving a positive predictive value (PPV) of 95.9% (95% CI: 88.5–99.1%), with a theoretical PPV range of 92.1–96.1%. The median LLR score in the 70 TP cases was 146, with a maximum of 726.74 and a minimum of 8.7. The LLR scores for the three FP cases were 2.88, 3.58, and 2.75, which are very close to the cut-off value for trisomy 21 (2.5). The mosaic ratios for these three cases were 39%, 24%, and 78%, and the FFs were 5%, 8%, and 4%. Of the three cases without diagnostic testing, one rejected amniocentesis, and two had a miscarriage. The patient who rejected amniocentesis had a normal gestation with a healthy newborn; no further follow-up was available. This patient had a low LLR of 3.17 and an FF of 4%, which may explain this likely discordant result.

Our study had an SPR of 0.35% for trisomy 18 (21/5998). Nineteen of the trisomy 18 cases were from singleton pregnancies, and two were from VT pregnancies. The FF range for trisomy 18 cases was 4–16%. Fourteen cases were confirmed to be TPs and four were FPs, giving a PPV of 77.8 (95% CI: 50.1–93.2%), with a theoretical PPV range of 66.7–81.0%. The median LLR score in the 14 TP cases was 129.95, with a maximum of 2210.54 and a minimum of 23.28. One of the four FP cases had a low LLR of 3.36, a mosaic ratio of 32%, and an FF of 6%. The LLR cut-off value for trisomy 18 is 3.0, so this value may explain the discordant result. The other two FP cases were from VT pregnancies. One of these VT cases had the blood draw for cfDNA screening carried out at 17 weeks of gestation, with a suspected fetal demise at 16 weeks of gestation. The LLR in this case was 24.53, with an FF of 9% and a mosaic ratio of 24%. For the other VT case, cfDNA screening was carried out at 14 weeks of gestation; however, at 13 and 5 weeks, one of the two embryos had no cardiac activity. The LLR in this case was 42.06, with an FF of 13% and a mosaic ratio of 22%. In the fourth FP case, trisomy 18 was not confirmed in the fetus after amniocentesis, but the newborn had bilateral heterotopias, arachnoid cysts, and congenital heart disease. This case had a very high LLR of 286, a mosaic ratio of 79.9%, and an FF of 11%; however, postnatal genetic studies performed were normal. Of the three trisomy 18 cases without diagnostic testing, one had a miscarriage (it was noted that the fetus had cystic hygroma; LLR of 98, a mosaic ratio of 53%, and an FF of 5%), and two rejected amniocentesis. The first one had an elective termination due to a polymalformed fetus with holoprosencephaly (LLR of 61.92, a mosaic ratio of 80%, and FF 6%), and the second one had a normal gestation and delivery (this case had an LLR of 4.8 close to the cut-off value, a mosaic ratio of 34%, and an FF of 6%).

The SPR for trisomy 13 in our study was 0.12% (7/5998). Of the seven trisomy 13 cases, six were singleton and one was from a VT pregnancy. FFs for these cases ranged from 6 to 15%. Four of the trisomy 13 cases were confirmed as TPs and two were FPs, giving a PPV of 66.7% (95% CI: 22.3–95.7%), with a theoretical PPV range of 57.1–71.4%. The median LLR score in the four TP cases was 134.02, with a maximum of 146.8 and a minimum of 82. The two FP cases had LLRs of 6.9 and 3.08 (the cut-off for trisomy 13 is 3.0), mosaic ratios of 27% and 24%, and FFs of 15% and 6%. The patient who chose not to undergo diagnostic testing had a VT pregnancy. For this patient, a demised twin was noted at 8 weeks of gestation, and the initial blood draw for cfDNA screening was carried out at 12 weeks. This case had an initial LLR of 25.57, a mosaic ratio of 43%, and an FF of 7%. However, cfDNA screening was repeated in another laboratory later in the pregnancy, and a normal result was returned, and clinical follow-up determined a healthy newborn. 

Twenty-nine patients in our cohort had an SCA call following cfDNA screening (SPR of 0.48%). All cases were from singleton pregnancies that underwent genome-wide cfDNA screening with an FF range of 4–20%. Overall, there were 15 cases with X0 (monosomy X; Turner syndrome), 4 cases with XXY (Klinefelter syndrome), 4 cases with XYY (Double Y syndrome), and 6 cases with XXX (Triple X syndrome). Of the 15 cases with monosomy X (X0), 13 underwent diagnostic testing. Of these 13 cases, 5 were TP (three of which were mosaic), 7 were FPs, and 1 case had a discordant result (XXX) following amniocentesis. However, for the other SCA cases (XXY, XYY, and XXX), 9 cases underwent diagnostic testing, and all of them were concordant with the cfDNA screening result (2 XYY, 2 XXY, and 5 XXX). The PPV for SCA cases was 63.6% (95% CI: 45.1–86.1%), with a theoretical PPV range of 48.3–72.4%. Details of these 29 cases are shown in Table S1. 

**Figure 2:**
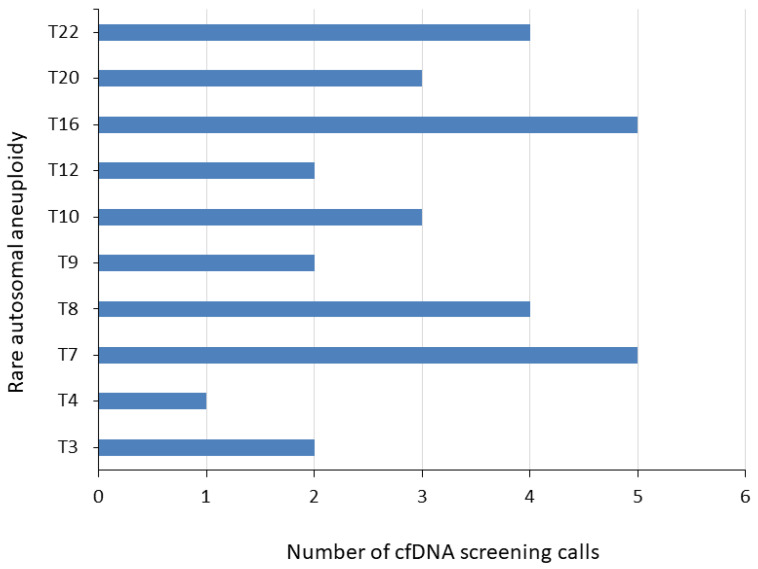
Overview of RAA calls by prenatal cfDNA screening. RAA, rare autosomal aneuploidy; cf, cell-free; T, trisomy.

As noted above, 31 cases in our study had a high-risk RAA result following cfDNA screening (SPR of 0.52%); see Table S2. Thirty cases were singleton and one was from a VT pregnancy with an FF range of 3–20%. Figure 2 provides an overview of the different RAAs, with trisomy 7 (n = 5) and trisomy 16 (n = 5) being the most common RAAs in the cohort. Twenty-eight cases underwent diagnostic testing, with three confirmed to be concordant with the cfDNA screening result, giving a PPV of 10.7% (3/28, 95% CI: 2.3–28.2%), with a theoretical PPV range of 9.7–19.4%. One of the three concordant cases was a trisomy 16 case that was noted as being likely mosaic with a karyotype of 47, XX, + 16[1]/46, XX[84] following amniocentesis; this patient experienced a miscarriage. Another concordant case (trisomy 22) was confirmed as 16% mosaic using amniocentesis and POC analysis by FISH [nuc ish(ABL1x2,BCRx3)[32/200]] and karyotyping [47,XX, + 22(9)]. The third concordant case (trisomy 16) was confirmed as 14% mosaic following amniocentesis with array [arr(16) × 3(0.14)]; growth restriction and preeclampsia were detected during the pregnancy, and a caesarean section was performed at 36 and 5 weeks due to severe FGR. A postnatal genetic study was performed, resulting in a maternal partial uniparental isodisomy, [arr[GRCh37] 16p13.3p12.3(94808_18843684) × 2 hmz].

Of these 28 RAA cases, 25 patients had a result on diagnostic testing that was discordant with the cfDNA screening result. One patient who underwent amniocentesis also had a discordant uniparental disomy (UPD) in chromosome 20. Another discordant case had a high risk of trisomy 9. In this case, a postnatal genetic study noted a normal newborn karyotype, and the FP NIPT result was due to maternal mosaic trisomy 9. Some of the patients with discordant RAA results presented adverse outcomes during the pregnancy, which included gestational diabetes, preeclampsia, FGR, and miscarriage. Postnatal complications included reduced birth weight and major congenital structural abnormalities. An overview of perinatal outcomes for RAA cases is provided in Table 4, with further details in Table S2. More information is provided in Supplementary Appendix A.

There were 31 CNV cases in our study cohort (SPR of 0.52%), which were found on 13 different chromosomes (see Table S3); FFs ranged from 3 to 18%. Twenty-nine cases were singleton, one was from a twin, and one was from a VT pregnancy. Twenty-eight patients underwent confirmatory diagnostic testing. One of the remaining patients rejected invasive testing, one had an elective termination (the fetus showed at 12 weeks of gestation a hydrops with a nuchal translucency of 4.2 mm), and one patient underwent a repeat cfDNA screening with a low-risk result. Of the 28 patients that had diagnostic testing, three were confirmed to be concordant, giving a PPV of 10.7% (95% CI: 2.3–28.2%) with a theoretical PPV range of 9.7–19.4%.

Nine patients (eight singletons and one VT pregnancy) in our study had multiple chromosomal anomalies (SPR of 0.15%) and had an FF range of 5–17%. As shown in Table S4, six patients had a normal result by amniocentesis, although one of these patients suspected maternal malignancy due to more than two aberrations being observed following sequencing. This patient was diagnosed with an advanced gastric carcinoma, and the pregnancy was terminated. In the other three cases, two chromosomal anomalies were detected, but only one was confirmed after amniocentesis or POC tissue.

There were 122 twin pregnancies and 26 VT pregnancies in our study cohort. Three of the twins and six of the VT pregnancies had a high-risk cfDNA screening call; all nine affected pregnancies had genome-wide cfDNA screening. Details of these nine affected cases are described above and shown in Table S5. Seven cases underwent diagnostic testing, with two twin cases and four VT cases obtaining an FP result (one of the FP VT pregnancies had a fetus with a structural heart anomaly). The other twin case had a TP result, with trisomy 21 confirmed in one of the twins.

## 4. Discussion

The present study describes our clinical experience with genome-wide cfDNA screening in a large cohort of pregnant patients over a three-year period. Our results showed strong performance for the detection of fetal chromosomal anomalies, with a low no-call rate of 0.43%. High PPV was noted for trisomy 21, with similar values as the TRIDENT study results [18]. However, PPVs for trisomy 18 and 13 were lower than those described previously [18], and we consider that this is probably due to several reasons. One reason is the presence of VT pregnancies. It is noted that the PPV for trisomy 18 would increase to 87.5% if we excluded VT cases from the analysis. The second reason is the presence of confined placental mosaicism (CPM), which is more present in these trisomies than in trisomy 21 [19], and in our study, the presence of CPM cannot be determined using fetal amniotic fluid as confirmatory diagnostic testing. This in turn would have resulted in a higher PPV for those cases. Finally, the third reason is the LLR threshold value used to establish the high-risk call. In common trisomies, most FP cases had a low LLR value close to the threshold. These data suggest that clinical laboratories must establish their own LLR threshold value in order to decrease the number of FP and increase the PPV of these common aneuploidies and LLR value could be a useful tool to be considered in NIPT reports. In addition, the optimization of the LLR value could reduce invasive tests. However, we noted that some FP cases with high LLR values could also probably be low-level mosaic undetected; therefore, clinical follow-up and postnatal studies in other tissues should be performed (i.e., postnatal karyotype or FISH in skin biopsy).

Cell-free DNA-based prenatal testing in pregnancies with SCAs has lower sensitivities and specificities as compared to testing for the common fetal autosomal trisomies [20]. It has been reported that in women of advanced reproductive age, there is a gradual and preferential loss of the inactive X chromosome in blood cells, thus converting their blood karyotype from XX to an XO/XX mosaic [21,22]. This maternal mosaicism may be a contributor to discordant NIPT results, especially for X0 cases, whose PPVs in our cohort are lower than in other SCA cases (XXY, XYY, and XXX) most probably due to this maternal X chromosome loss.

In our study, 0.52% of patients screened positive for the presence of an RAA; previous studies have noted SPRs of 0.12–1.1% [23,24]. The most common RAAs in our cohort were trisomy 7 and trisomy 16. Trisomy 7 is the most frequently detected RAA in other genome-wide cfDNA screening studies [9,14,18,23,25], and trisomy 16 is thought to be the most common RAA in adverse pregnancy outcomes [15,26]. Our calculated PPV for RAAs was 10.7%, which is similar to the 11.46% pooled PPV noted in a recent meta-analysis of studies with rare autosomal trisomies [27]. One potential reason for the low PPV typically observed in RAA cases, apart from its low prevalence, may be due to the type of diagnostic testing performed. As noted in previous studies [9,15], many patients with an RAA call on cfDNA screening may have CPM; however, in our study, almost all RAA cases that underwent diagnostic testing (27/28, 96.4%) had amniocentesis with no placental testing performed. Therefore, it is possible that some of the discordant results may have been CPM cases, which would have resulted in a higher PPV.

Importantly, numerous studies have shown that CPM RAA cases are associated with adverse pregnancy outcomes and perinatal complications, including preeclampsia, FGR, preterm birth, reduced birth weight, and structural fetal anomalies [9,15,28]. In these cases, individualized clinical management of these patients is suggested [9,15]. In our study, 17 RAA cases had adverse perinatal complications or outcomes. Focusing on the two most common RAAs in our study, reduced birth weight was noted in two/five cases of trisomy 7, with one of these cases also having preeclampsia; four/five cases of trisomy 16 had FGR, preeclampsia, gestational diabetes, and spontaneous abortions. However, the number of cases in our study was not large enough to draw any definitive conclusions regarding the impact of individual RAAs on patient outcomes. TRIDENT study in the Netherlands noted perinatal complications in some cases with CPM trisomy 7 and trisomy 16, including preeclampsia, pregnancy-induced hypertension, and congenital structural abnormalities [15].

Our SPR for CNV cases was 0.52%, which is higher than that noted in some other previous studies [14,18], but it is similar to the 0.56% SPR in the study by Rafalko et al. [12]. Based on cases with diagnostic testing (28/31), our PPV for CNVs was 10.7%. Nevertheless, previous publications noted higher PPVs of 44.1–74.2% [12,14,15], contrary to a PPV of 19.1% described by Raymond et al. [29]. Our results disagree with the literature due to the limited number of CNVs or other reasons, including CPM, the presence of maternal CNVs, and maternal malignancies. We detected a recurrent specific deletion starting at 10q25 in three pregnancies, which has been previously described [30]; this is considered an FP caused by a maternal mosaic deletion associated with an expanded fragile site (FRA10B). Another FP case was due to maternal malignancy, in which the patient was diagnosed with advanced gastric carcinoma during gestation. Previous studies have shown that maternal malignancies can be a source of FP NIPT results [31,32].

In our study, 13 of the 31 CNV patients had adverse perinatal complications or outcomes. Clinical consequences are variable and likely depend on several factors, including the size of the CNV and the region in which it occurs.

It should be noted that the PPVs for both RAAs and CNVs in our study are still much higher (10.7%) than the PPVs for trisomies 21 and 18 using conventional serum screening (3.4%) [33]. It is important to consider performing genome-wide NIPT to detect these less common alterations and improve the management of pregnancies. In addition, screening for these additional anomalies does not result in a significant increase in the number of invasive techniques performed due to the low frequency of these rarer alterations. However, contrary to common trisomies, the LLR value has not been demonstrated to be a useful tool in RAA and CNV cases. Therefore, it is necessary to increase the number of RAA and CNV cases to optimize the LLR threshold before reaching clinical conclusions.

The strength of our study was that clinical follow-up was available for most high-risk cases, allowing us to determine PPVs for each of the common trisomies, SCAs, RAAs, and CNVs. All samples were analyzed in the same hospital laboratory.

Nevertheless, we suggest performing placental testing in RAA and CNV cases in order to determine if any of them were CPM cases and their impact on the PPV. Overall, the limited number of high-risk cases in our cohort had a direct impact on the final PPV values, which were somewhat lower than expected, and also precluded us from drawing definitive conclusions regarding the impact of these anomalies on patient outcomes. It is notable that there could be a bias in our results due to the different cFTS risk score used in our cohort (up to 270 during the first 10 months, then expanded up to 1000).

## 5. Conclusions

In conclusion, our study showed strong performance for genome-wide cfDNA screening in a large cohort of pregnant patients in Spain. We found that screening for less common chromosomal anomalies could identify patients at risk of perinatal complications who may require additional screening or clinical management during pregnancy. In addition, the number of CNV and RAA cases is higher than some common trisomies if we consider them as individual entities.

Currently, professional medical societies do not recommend genome-wide cfDNA screening in general pregnancy populations due to insufficient clinical evidence, though our data show the importance of considering the screening for these additional genetic findings. Future steps would include carrying out more comprehensive follow-up on all patients, including placental testing for RAA cases.

## Figures and Tables

**Table 1:** Demographics of pregnant patients (n = 5998).

Variable	Mean + SD	Median	Range
Maternal age, years	35.48 ± 5.18	36	18–53
Gestational age, weeks	13.50 ± 2.34	13.99	10–37
Body mass index (BMI)	25.25 ± 5.07	24.17	14.69–54.20

**Table 2:** Testing indications of the study cohort (n = 5998).

Test Indication	n (%)
High-risk first-trimester screening	4677 (77.98)
High-risk second-trimester screening	692 (11.54)
No first-trimester screening	217 (3.62)
Previously affected pregnancy	171 (2.85)
X-linked disease	34 (0.57)
Parental translocation carrier	9 (0.15)
Other ^a^	154 (2.57)
Unknown/not specified	44 (0.73)

n, number of patients. ^a^ Vanishing twin; triple gestations (patients with a fetal demise that are included in the vanishing twin cohort); patients with advanced maternal age; miscarriage; placenta dysfunction (included patients with abnormal serum screening β-hCG/PAPP-A) and ultrasound anomalies (included high nuchal translucency). Β-hCG, β human chorionic gonadotropin; PAPP-A, pregnancy associated plasma protein-A.

**Table 3:** Cell-free DNA screening results and concordance with clinical outcomes for common trisomies and sex chromosome aneuploidies.

cfDNA Screening Result	Number	No Diagnostic Testing	True Positive	False Positive	PPV, % (95% CI)
T21	76	3	70	3	95.9 (88.5–99.1)
T18	21	3	14	4	77.8 (50.1–93.2)
T13	7	1	4	2	66.7 (22.3–95.7)
SCAs	29	7	14	8 *	63.6 (45.1–86.1)

PPV, positive predictive value; CI, confidence interval; T, trisomy; SCAs, sex chromosome aneuploidies. * One case had a cfDNA screening result of X0, but clinical follow-up determined that it was an XXX case. We have included this case as a false positive in the PPV calculation.

**Table 4:** Characteristics and clinical outcomes of cases with a high-risk NIPT result for an RAA or CNV.

	RAA Cases (n = 26)	CNV Cases (n = 28)
**Pregnancy complications (%)**		
Gestational hypertension	0 (0)	2 (7.1)
Preeclampsia (or suspicion of preeclampsia)	4 (15.4)	1 (3.6)
Chronic hypertension	1 (3.9)	1 (3.6)
Gestational diabetes	4 (15.4)	4 (14.3)
Fetal growth restriction	6 (23.1)	5 (17.9)
Placental alterations	0 (0)	3 (10.7)
**Table Pregnancy outcomes (%)**		
Spontaneous abortion (<20 wk)	3 (11.5)	2 (7.1) ^a^
Intrauterine fetal demise (>20 wk)	0 (0)	2 (7.1)
Elective termination	0 (0)	2 (7.1)
Preterm birth (<37 wk)	3 (11.5)	3 (10.7)
Emergency C-section before 34 wk	1 (3.9)	1 (3.6)
Emergency C-section between 34 and 37 wk	2 (7.7)	0 (0)
Emergency C-section >37 wk	2 (7.7)	6 (21.4)
**Neonatal outcomes (%)**		
5-min Apgar score < 7	1 (3.9)	0 (0)
Reduced birth weight	7 (26.9)	1 (3.6)
Admission to the NICU	3 (11.5)	2 (7.1)
Neonatal death	1 (3.9)	1 (3.6)
Major congenital structural abnormalities ^b^	3 (11.5)	4 (14.3)

Values are provided as number of cases (percentage). Patient characteristics are based on the entire RAA or CNV cohort. Complete follow-up was available for most of the listed cases. Not all cases are exclusive. RAA, rare autosomal aneuploidy; CNV, copy-number variant; n, number; wk, weeks; min, minute; NICU, neonatal intensive care unit. ^a^ One of these cases involved the spontaneous abortion of both twins in a twin pregnancy. ^b^ Detected by ultrasound scan, visual inspection at birth, autopsy, or at longer-term follow-up.

## Data Availability

The datasets generated during the current study are available from the corresponding author upon reasonable request.

## Supplementary Materials

The following supporting information can be downloaded at https://www.mdpi.com/article/10.3390/genes15050568/s1. Supplementary Appendix A, Table S1: Cell-free DNA screening results and concordance with clinical outcomes for SCA cases, Table S2: Cell-free DNA screening results and clinical outcomes for RAA cases, Table S3: Cell-free DNA screening results and clinical outcomes for CNV cases, Table S4: Cell-free DNA screening results and clinical outcomes for multiple anomalies cases, Table S5: Overview of high-risk twin pregnancies.
