# Supplementary material for: Prenatal Genome-Wide Cell-Free DNA Screening: Three Years of Clinical Experience in a Hospital Prenatal Diagnostic Unit in Spain
Source: Genes (Basel). 2024 Apr 28;15(5):568. doi: 10.3390/genes15050568 (PMC11121632; doi:10.3390/genes15050568)
Supplement: Supplementary file 1 [file genes-15-00568-s001.zip › genes-2972921-supplementary.pdf]

## SUPPLEMENTARY MATERIALS

# Prenatal Genome-Wide Cell-free DNA Screening: Three Years of Clinical Experience in a Hospital Prenatal Diagnostic Unit in Spain

Laia Pedrola Vidal[1], Mónica Roselló Piera[1,*], Carla Martín-Grau[2], Juan S. Rubio Moll[3], Rosa Gómez Portero[3], Beatriz Marcos Puig[3], Jose V. Cervera Zamora[1], Ramiro Quiroga[3] and Carmen Orellana Alonso[1]

[1]Genetics Unit, Hospital Universitari i Politècnic La Fe, Valencia, Spain
[2]Translational Genetics Research Group, Instituto de Investigación Sanitaria La Fe (IISLAFE), Valencia, Spain
[3]Obstetrics and Gynecology Unit, Hospital Universitari i Politècnic La Fe, Valencia, Spain
[*]Correspondence: rosello_mpi@gva.es

| Table of Contents | Page |
|---|---|
| Supplementary Appendix A | 2 |
| Table S1. Cell-free DNA screening results and concordance with clinical outcomes for SCA cases. | 3 |
| Table S2. Cell-free DNA screening results and clinical outcomes for RAA cases. | 4 |
| Table S3. Cell-free DNA screening results and clinical outcomes for CNV cases. | 7 |
| Table S4. Cell-free DNA screening results and clinical outcomes for multiple anomalies cases. | 10 |
| Table S5. Overview of high-risk twin pregnancies. | 11 |

**Supplementary Appendix A**

One case in the rare autosomal aneuploidies (RAA) cohort was a trisomy 20 case that had an initial low-risk result with fetal sex XY on cell-free DNA (cfDNA) analysis. An ultrasound scan noted a female fetus, and a second cfDNA analysis was performed giving a high-risk result for trisomy 20 (fetal sex XY). In this case the log likelihood ratio (LLR) was 16.5 (close to the reference 10.6), the mosaic ratio was 25%, and the fetal fraction (FF) was 14%. An amniocentesis with array was performed in a private center with the karyotype result mos 46,X,+mar[25]/45,X[5] and the array showed two different deletions on chromosome Y (and trisomy 20 was discordant). One of these was a terminal deletion of region Yp11.32p11.2 (chrY: 118552-3082444; Hg19) of 2.96 Mb with 28 OMIM® genes (118552-3082444), and this deletion affected the *SHOX* and *SRY* genes: arr [GRCh37]Yp11.32p11.2(118,552-3,082,444)x0,Yq11.221q12(18,292,329-59,336,104)x0. The other deletion was a terminal deletion of region Yq11.221q12 (chrY: 18292329-59336104; Hg19) of 41.04 Mb encompassing 27 OMIM® genes (18292329-59336104). With regards to the first deletion, pathogenic deletions or point mutations of the *SHOX* (short stature homeobox) gene give rise to its haploinsufficiency. The phenotypic spectrum ranges from Leri-Weill dyschondrosteosis (with the classic clinical triad of short stature, mesomelia, and Madelung deformity) at the severe end of the spectrum to nonspecific short stature at the mild end of the spectrum. The deletion of the *SRY* gene in a patient with the male genetic sex is associated with complete gonadal dysgenesis or Swyer syndrome (OMIM® 400044). It is a disorder of sexual development associated with anomalies in gonadal development. Individuals with 46,XY gonadal dysgenesis may present from ambiguous genitalia to phenotypically female genitalia; however, they do not develop secondary sexual characteristics at puberty and have primary amenorrhea. The gonads are usually made up of fibrous tissue and are associated with an increased risk of developing tumors (mainly dysgerminomas), they usually present a uterus and fallopian tubes, and the external genitalia are female. This deletion of the *SRY* gene explains the discrepancy observed between the cfDNA result (male) and ultrasound sex (female). With regards to the second deletion, deletions of the long arm of the Y chromosome (Yq) represent one of the known causes of failure in spermatogenesis. It is produced by the loss of one or more of three loci called azoospermia factor (AZF)a, AZFb, and AZFc causing infertility in males. In general, the symptoms associated with the detected chromosomal alteration (45,X/46,XY) are very similar to those observed in patients with Turner syndrome, although because it is a chromosomal mosaic, they may present greater variability of symptoms. The most characteristic symptoms presented by the majority of patients with complete Turner syndrome (chromosomal set 45,X in all cells) are short stature and primary amenorrhea, with ribbon gonads and absence of ovarian follicles. In this case, we would recommend consultation with a pediatric endocrinologist.

Abbreviations: Mb, megabase; OMIM®, Online Mendelian Inheritance in Man®.

**Table S1. Cell-free DNA screening results and concordance with clinical outcomes for SCA cases.**

| Case | GA, wk | FF, % | cfDNA Screening Result | Confirmatory Testing | Genetic Result |
|---|---|---|---|---|---|
| 1 | 12 | 16 | 45,X | Amniocentesis | 46,XX |
| 2 | 12 | 11 | 45,X | Amniocentesis | Concordant: 45,X |
| 3[a] | 12 | 6 | 45,X | No confirmatory testing | N/a |
| 4 | 13 | 13 | 45,X | Amniocentesis | 46,XX |
| 5 | 13 | 7 | 45,X | Amniocentesis | 46,XX |
| 6[a] | 14 | 15 | 45,X | CVS | Concordant: 45,X |
| 7 | 13 | 7 | 45,X | Amniocentesis | Concordant: 45,X[18%] |
| 8 | 12 | 15 | 45,X | Amniocentesis | 46,XX |
| 9[b] | 13 | 6 | 45,X | No confirmatory testing | N/a |
| 10 | 13 | 10 | 45,X | Amniocentesis | 46,XX |
| 11 | 12 | 7 | 45,X | Amniocentesis | 46,XX |
| 12 | 16 | 8 | 45,X | Amniocentesis | 46,XX |
| 13 | 13 | 4 | 45,X | Amniocentesis | Concordant: 45,X[60%] |
| 14[a] | 15 | 8 | 45,X | Amniocentesis | Discordant: XXX |
| 15 | 12 | 8 | 45,X | Amniocentesis | Concordant: 45,X |
| 16 | 13 | 10 | 47,XXY | No confirmatory testing | N/a |
| 17 | 13 | 12 | 47,XXY | Amniocentesis | Concordant: 47,XXY |
| 18 | 26 | 9 | 47,XXY | No confirmatory testing | N/a |
| 19 | 12 | 6 | 47,XXY | CVS | Concordant: 47,XXY |
| 20 | 12 | 11 | 47,XYY | Amniocentesis | Concordant: 47,XYY |
| 21 | 21 | 15 | 47,XYY | No confirmatory testing | N/a |
| 22 | 16 | 8 | 47,XYY | No confirmatory testing | N/a |
| 23 | 12 | 4 | 47,XYY | Amniocentesis | Concordant: 47,XYY |
| 24 | 14 | 11 | 47,XXX | Amniocentesis | Concordant: 47,XXX |
| 25 | 13 | 20 | 47,XXX | Amniocentesis | Concordant: 47,XXX |
| 26 | 13 | 11 | 47,XXX | Amniocentesis | Concordant: 47,XXX |
| 27 | 12 | 5 | 47,XXX | Amniocentesis | Concordant[c]: 47,XXX |
| 28 | 13 | 5 | 47,XXX | Amniocentesis | Concordant: 47,XXX |
| 29 | 14 | 7 | 47,XXX | No confirmatory testing | N/a |

In case of amniocentesis or chorionic villus sampling (CVS), some laboratory techniques are performed as quantitative fluorescent PCR (QF-PCR), karyotype or both. SCA, sex chromosome aneuploidies; GA, gestational age; wk, weeks; FF, fetal fraction; cfDNA, cell-free DNA; N/a, not available. [a]Patient had an elective termination. [b]Newborn died at 18 days of life. A homozygous deletion of the *GBE1* gene was detected associated with glycogenosis type IV. [c]arr[GRCh37] Xp22.33q28(168547_155233731)x3.

## Table S2. Cell-free DNA screening results and clinical outcomes for RAA cases.

| Case | GA, wk | FF, % | cfDNA Screening Result | Confirmatory Testing | Genetic Result | Pregnancy Outcomes | Birth Outcomes | LLR Threshold | LLR Sample | Mosaic Ratio (%) |
|---|---|---|---|---|---|---|---|---|---|---|
| 1 | 12 | 7 | T3 | Amniocentesis | 46,XX | Gestational diabetes | Normal | 5 | 65.67 | 84.32 |
| 2 | 13 | 5 | T3 | Amniocentesis | 46,XY | Normal | Normal | 5 | 12.51 | 37.69 |
| 3 | 14 | 10 | T4 | Amniocentesis | 46,XY | Obstetric metrorrhagia in the third trimester | Normal | 7 | 675.88 | 100 |
| 4 | 12 | 19 | T7 | Amniocentesis | 46,XY | PAPP-A was 0.18; late FGR (36 wk) | Emergency C-section at 36+5 wk; reduced birth weight (1,980g; 2nd percentile); neonatal hypoglycaemia | 6.6 | 2772.04 | 82 |
| 5 | 13 | 7 | T7 | Amniocentesis | 46,XX | Preeclampsia | Reduced birth weight at 38+2 wk (2,480g, 8th percentile) | 6.6 | 26.81 | 40 |
| 6 | 14 | 14 | T7 | Amniocentesis | 46,XY | Normal | Normal | 6.6 | 130 | 38 |
| 7 | 13 | 14 | T7 | Amniocentesis | 46,XX | Normal | Cerebral haemorrhage due to arteriovenous malformation | 6.6 | 876.46 | 82 |
| 8 | 13 | 5 | T7 | Amniocentesis | 46,XY | Normal | Normal | 6.6 | 12.77 | 36.89 |
| 9 | 13 | 11 | T8 | Amniocentesis | 46,XY | Normal | Normal | 6.6 | 17.12 | 21 |
| 10 | 12 | 9 | T8 | No confirmatory testing | N/a | N/a | N/a | 5.8 | 19.52 | 21.88 |
| 11 | 12 | 8 | T8 | Amniocentesis | 46,XY | Obstetric metrorrhagia and uterine hypertonus | Spontaneous preterm birth at 24+6 wk; low Apgar score; admission to NICU; neonatal death at 23 h | 5.8 | 130 | 38 |
| 12 | 12 | 5 | T8 | Amniocentesis | 46,XX | Suspicion of late preeclampsia | Normal | 5.8 | 100.19 | 100 |
| 13 | 13 | 5 | T9 | Amniocentesis | 46,XY | Preeclampsia; FGR before 32 wk | Delivery by C-section >37 wk; baby had short stature and short long bones; exome sequencing was performed with a homozygous VOUS in *NPR2* gene suggesting Aarskog syndrome | 8 | 157.72 | 100 |
| 14[a] | 16 | 15 | T9 | Postnatal | 46,XX | Fetal echogenic bowel and choroid plexus cysts. Mild preeclampsia and oligoamnios. | Normal | 8 | 609.88 | 6.2 |
| 15 | 13 | 5 | T10 | Amniocentesis | 46,XX | Normal | Reduced birth weight at 40+2 wk (2,730g, 5th percentile) | 8.8 | 41.32 | 62 |
| 16 | 12 | 12 | T10 | Amniocentesis | 46,XX | Normal | Normal | 8.8 | 168.41 | 44.43 |
| 17 | 12 | 7 | T10 | Amniocentesis | 46,XX | Normal | N/a | 8.8 | 74.59 | 51.44 |

| | | | | | | | | | | |
|---|---|---|---|---|---|---|---|---|---|---|
| 18 | 13 | 11 | T12 | Amniocentesis | 46,XY | Chronic hypertension | Emergency C-section at 30+4 wk; baby was admitted to the NICU with respiratory distress | 11.6 | 324.35 | 73.42 |
| 19 | 10 | 10 | T12 | CVS | 46,XY | Unknown | Emergency C-section >37 weeks | 11.6 | 15.8 | 19.74 |
| 20 | 12 | 20 | T16 | Amniocentesis | 46,XX | Normal | N/a | 10.7 | 966.76 | 71 |
| 21 | 12 | 5 | T16 | Amniocentesis | Concordant: 47,XX,+16[1]/46,XX[84] | Spontaneous abortion at 17 wk | N/a | 10.7 | N/a | N/a |
| 22 | 12 | 15 | T16 | Amniocentesis | 46,XX | Spontaneous abortion at 16 wk | Autopsy noted intestinal and hepatic gastroschisis | 10.7 | 889.47 | 94.15 |
| 23 | 11 | 11 | T16 | Amniocentesis | 46,XX | Hypothyroidism, gestational diabetes, isolated FGR <32 wk | Baby had mucocutaneous jaundice that did not require phototherapy and weight loss that required supplemental breastfeeding | 10.7 | 64.9 | 59.63 |
| 24[b] | 15 | 3 | T16 | Amniocentesis and postnatal | Concordant: [arr(16)x3(0,14)] and [arr[GRCh37] 16p13.3p12.3(94808_18843684)x2 hmz] | FGR and preeclampsia. Patient was followed until 36 wk but had delivery in a private center | Newborn was seen at our hospital at the age of 20 days (weight at birth: 1,840 g, 1st percentile); baby was followed at the hospital because of digestive problems, stools with mucus, irritability after feedings. | 10.7 | 14.07 | 100 |
| 25 | 12 | 15 | T20 | Amniocentesis | 46,XY | Late FGR (36 wk); premature rupture of membranes | Spontaneous preterm birth at 37+2 wk; low birth weight (2,580g, 16th percentile) | 10.6 | 428.59 | 86.6 |
| 26 | 20 | 14 | T20 | Amniocentesis | arr[GRCh37] Yp11.32p11.2 (118,552-3,082,444)x0,Yq11.221q12 (18,292,329-59,336,104)x0 | Normal | Normal | 10.6 | 369.01 | 100 |
| 27 | 12 | 6 | T20 | Amniocentesis | 46,XY | Gestational diabetes | Low birth weight (2,760g, 10th percentile) | 10.6 | 30.37 | 63.82 |
| 28 | 13 | 7 | T22 | Amniocentesis | 46,XY | Normal | Preterm birth at 33+2 wk; baby was admitted to the NICU due to prematurity | 13.5 | 67.91 | 75 |
| 29 | 12 | 8 | T22 | Amniocentesis | 46,XY | Gestational diabetes; Isolated FGR <32 wk | Emergency C-section at 37 wk; very low birth weight (1,750g, <1st percentile) | 13.5 | 84.23 | 72.9 |
| 30 | 12 | 5 | T22 | Amniocentesis and POC | Concordant: 47,XX,+22(9) | Spontaneous abortion at 16 wk | N/a | 13.5 | 45.78 | 100 |
| 31 | 12 | 11 | T22 | Amniocentesis | 46,XY | Normal | Normal | 13.5 | 15.3 | 36.11 |

In case of amniocentesis, chorionic villus sampling (CVS), products of conception (POC), or postnatal studies, some laboratory techniques are performed as quantitative fluorescent PCR (QF-PCR), karyotype, or both and array. RAA, rare autosomal aneuploidies; GA, gestational age; wk, weeks; FF, fetal fraction; cfDNA, cell-free DNA; LLR, log likelihood ratio; T, trisomy; PAPP-A, pregnancy associated plasma protein-A; FGR, fetal growth restriction; g, grams; C-section, caesarean section; N/a, not available; NICU, neonatal intensive care unit; h, hours; VOUS, variant of uncertain significance. [a]Maternal mosaic of trisomy 9. [b]Trisomy 16 confirmed as 14% mosaic following amniocentesis with array [arr(16)x3(0,14)]. Postnatal genetic study was performed in peripheral blood resulting a maternal partial uniparental isodisomy [arr[GRCh37] 16p13.3p12.3(94808_18843684)x2hmz].

**Table S3. Cell-free DNA screening results and clinical outcomes for CNV cases.**

| Case | GA, wk | FF, % | cfDNA Screening Result | Confirmatory Testing | Genetic Result | Pregnancy Outcomes | Birth Outcomes |
|---|---|---|---|---|---|---|---|
| 1 | 12 | 8 | dup(1)(q21.1q32.2) | Amniocentesis | 46,XX | Normal | Normal |
| 2 | 13 | 5 | dup(1)(p22.3q25.3) | No confirmatory testing | N/a | Gestational diabetes | Emergency C-section >37wk due to premature rupture of membranes |
| 3 | 16 | 6 | dup(1)(p32.3q31.2) | Amniocentesis | 46,XX | Normal | Emergency C-section at 32+2 wk due to threatened premature birth; admission to NICU due to prematurity; baby had slight retrognathia; inguinal hernia operated on 4 months post-birth; baby girl was born at 32+2 wk, weight:1,960 g (72nd percentile). |
| 4 | 12 | 7 | dup(1)(p35.1p31.3) | Amniocentesis | 46,XY | Placental hydrops; spontaneous abortion at 16 wk | N/a |
| 5 | 14 | 4 | dup(1)(p36.22p33) | Amniocentesis | 46,XX | Normal | Normal |
| 6 | 13 | 14 | del(5)(q14.3q32) | Amniocentesis | 46,XY | Pregnancy in progress; ultrasound scan at 32 wk noted fetal ventricular asymmetry which was confirmed with an MRI | N/a |
| 7[a] | 14 | 4 | del(5)(p15.33p13.3) | Amniocentesis | 46,XY but array showed LOH chr 5 arr[GRCh38] 5p15.33p13.3(113462_30725947)x2 hmz | Preeclampsia; isolated FGR <32 wk | Emergency C-section >37 wk; baby was admitted to the NICU with early hypoglycemia; neonatal death occurred at 21 days; baby had microcephalus and mild right pyelectasis |
| 8[b] | 12 | 3 | dup(5)(p15.1p13.3) | No confirmatory testing | N/a | Gestational diabetes | Normal |
| 9[c] | 15 | 4 | del(7)(q21.11q31.32) | Amniocentesis | 46,XX | Gestational diabetes | Preterm birth |
| 10 | 12 | 6 | del(8)(p23.3p22) | Postnatal | 46,XY | Normal | Normal |
| 11 | 12 | 12 | del(8)(p23.3p11.21) | Amniocentesis | 46,XX | Gestational hypertension; late FGR (37 wk) | Normal |
| 12 | 18 | 16 | dup(9)(p24.3p13.1) | Amniocentesis | 46,XX | Chronic hypertension; isolated FGR <32 wk | Emergency C-section after 37 wk; reduced birth weight for 37+1 wk (1,950g, 1st percentile) |

| | | | | | | | |
|---|---|---|---|---|---|---|---|
| 13 | 18 | 10 | dup(10)(q11.21q26.13) | Amniocentesis | 46,XY | Gestational diabetes; intrauterine fetal demise at 28+3 wk | Weight was 836g; no autopsy was carried out but an anatomic pathology exam noted a mature placenta with signs of chorioamnionitis |
| 14 | 13 | 18 | dup(10)(p15.3q11.21) | Amniocentesis | 46,XX | Normal | Spontaneous preterm birth at 36+2 wk |
| 15 | 13 | 3 | del(10)(p15.3p12.31) | Amniocentesis | 46,XX | Severe FGR in one twin; spontaneous abortion of both twins at 19+3 wk | Pathological anatomy noted hydropic fetuses without congenital malformations |
| 16 | 13 | 7 | del(10)(q25.1q26.3) | Amniocentesis | 46,XX | Normal | Normal |
| 17 | 12 | 9 | del(10)(q25.2q26.3) | Amniocentesis | 46,XY | Normal | Normal |
| 18 | 12 | 6 | del(10)(q25.2q26.3) | Amniocentesis | 46,XX | Pregnancy in progress | N/a |
| 19[c] | 12 | 7 | del(13)(q12.11q12.2) | Amniocentesis | 46,XX | Gestational hypertension | Emergency C-section after 37 wk |
| 20 | 13 | 7 | del(13)(q13.3q14.3) | Amniocentesis | Concordant: 46,XY, del(13)(q13.3q14.3) | Termination of pregnancy | N/a |
| 21 | 14 | 7 | dup(13)(q14.3q21.33) | Amniocentesis | 46,XY | Normal | Normal |
| 22 | 13 | 8 | dup(13)(q14.3q34) | Amniocentesis | 46,XY | During gestation, ultrasound scan revealed mild bilateral renal pyelectasia | N/a |
| 23 | 13 | 4 | del(13)(q31.1q33.3) | Amniocentesis | 46,XY | Normal | Normal |
| 24 | 17 | 14 | del(13)(q31.1q31.3) | Amniocentesis | Concordant: 46,XX, del(13)(q31.1q31.3) | FGR | A baby girl was born who developed alterations in the carpus and tarsus with anomalous phalanges |
| 25 | 12 | 8 | del(14)(q31.1q32.33) | No confirmatory testing | N/a | Ultrasound at 12 wk noted fetal hydrops and an nuchal translucency of 4.2; termination of pregnancy at 15 wk | Autopsy detected small saddle nose, accentuated retrognathia, and low implantation of the auricle but no major malformations were detected |
| 26 | 14 | 18 | dup(15)(q14q26.3) | Amniocentesis | 46,XY | Normal | Normal |
| 27 | 12 | 11 | del(18)(p11.32p11.22) | CVS | Concordant: 46,XX,del(18)(p11.32p11.22) | Intrauterine fetal demise | N/a |
| 28 | 14 | 11 | dup(20)(p13q11.21) | Amniocentesis | 46,XY | Normal | Normal |
| 29 | 13 | 12 | dup(21)(q21.1q21.3) | Amniocentesis | 46,XX | Normal | Emergency C-section >37 wk |
| 30 | 13 | 10 | dup(21)(q21.1q22.3) | Amniocentesis | 46,XY | Normal | Emergency C-section >37wk |
| 31 | 12 | 15 | dup(22)(q11.21q13.31) | Amniocentesis | 46,XX | Metrorrhagia in the third trimester | Spontaneous preterm birth at 36+3 wk |

In case of amniocentesis or chorionic villus sampling (CVS), some laboratory techniques are performed as quantitative fluorescent PCR (QF-PCR), karyotype or both. CNV, copy-number variation; GA, gestational age; wk, weeks; FF, fetal fraction; cfDNA, cell-free DNA; del, deletion; dup, duplication; N/a, not available; C-section, caesarean section; NICU, neonatal intensive care unit; g, grams; MRI, magnetic resonance imaging; LOH, loss of heterozygosity; FGR, fetal growth restriction. [a]The patient had a controlled pregnancy and gave birth to a child with early hypoglycemia. At 24 hours of life, he presented hyperbilirubinemia and phototherapy was maintained for 2 days. The newborn also presented a

microcephaly but brain ultrasound scan was normal. Following neonatal death at 21 days, a clinical exome study was performed and was not informative. [b]cfDNA screening was repeated and a low-risk result was obtained. [c]Follow-up of newborn in another hospital.

## Table S4. Cell-free DNA screening results and clinical outcomes for multiple anomalies cases.

| Case | GA, wk | FF, % | cfDNA Screening Result | Confirmatory Testing | Genetic Result | Pregnancy Outcomes | Birth Outcomes |
|---|---|---|---|---|---|---|---|
| 1 | 14 | 8 | T4; dup(12)(q13.13q24.32) | Amniocentesis | 46,XY | Normal | Normal |
| 2 | 13 | 5 | T13;<br>T21 | Amniocentesis | 47,XY,+13 | Termination of pregnancy | N/a |
| 3 | 12 | 12 | T7;<br>T21 | Amniocentesis | 47,XX,+21 | Normal | Newborn compatible with Down syndrome phenotype |
| 4 | 12 | 11 | del(18)(q21.33q23); T22 | POC tissue | 46,XY,del(18)(q21.33q23)[a] | Termination of pregnancy | N/a |
| 5 | 13 | 9 | T12;<br>T15 | Amniocentesis | 46,XY | Fetal structural cardiopathy leading to hospitalization; FGR <32 wk; premature abruption of a normally inserted placenta/premature rupture of membranes | Emergency C-section at 26 wk; baby admitted to NICU due to prematurity; baby had congenital cardiopathy |
| 6[b] | 12 | 10 | T7;<br>T13 | Amniocentesis | 46,XX | Normal | N/a |
| 7[c] | 22 | 17 | dup(1)(p36.11p34.1); MX | Amniocentesis | 46,XX | Normal | Normal |
| 8[d] | 12 | 9 | del(1)(q42.12q42.3); dup(1)(q31.1q31.3) | Amniocentesis | 46,XX | Pregnancy ongoing (at 22 wk is normal) | N/a |
| 9[e] | 12 | 11 | 1st cfDNA screen:<br>T2; M4;<br>dup(7)(p22.1p15.2); T8;<br>M9<br>2nd cfDNA screen:<br>dup(2)(p21p16.2);<br>del(3)(p14.2p13); M4;<br>del(5)(q23.1q31.2);<br>del(6)(p12.3q27);<br>T7; T8; M9; M10; T11; M12; M13; M14; M15; M17; T18; T20; M21; T22 | Amniocentesis | 46,XX or 46,XY | Termination of pregnancy | N/a |

In case of amniocentesis or products of conception (POC), some laboratory techniques are performed as quantitative fluorescent PCR (QF-PCR), karyotype, or both. GA, gestational age; wk, weeks; FF, fetal fraction; cfDNA, cell-free DNA; T, trisomy; M, monosomy; del, deletion; dup, duplication; N/a, not available; FGR, fetal growth restriction; NICU, neonatal intensive care unit; C-section, caesarean section; Mb, megabases; kb, kilbases. [a]cfDNA screening called a deletion on chromosome 18 (q21.33- q23) that was 18.8 Mb. On array, a deletion on chromosome 18 was confirmed with a deletion size of 1.161 kb that was probably pathogenic. [b]Patient was followed up in another hospital. [c]At 36 wk the pregnancy was normal. Delivery was carried out in another center and no further follow-up was available. [d]At 22 wk gestation the pregnancy was normal. [e]Maternal malignancy case: at 23 weeks of gestation an advanced gastric carcinoma was detected and pregnancy was terminated.

## Table S5. Overview of high-risk twin pregnancies.

| Case | Type of Pregnancy | Maternal Age, yr | GA, wk | FF, % | cfDNA Screening Result | Clinical Follow-up |
|---|---|---|---|---|---|---|
| 1 | Twin | 29 | 13 | 8 | Trisomy 21 | Normal amniocentesis |
| 2 | Twin | 39 | 12 | 9 | Trisomy 21 | Trisomy 21 confirmed in one of the fetuses by amniocentesis |
| 3 | Twin | 30 | 13 | 3 | del(10)(p15.3p12.31) | Normal amniocentesis |
| 4[a] | Vanishing twin | 35 | 12 | 7 | Trisomy 13 | No diagnostic testing; normal pregnancy |
| 5[b] | Vanishing twin | 31 | 17 | 9 | Trisomy 18 | Normal amniocentesis |
| 6[c] | Vanishing twin | 43 | 14 | 13 | Trisomy 18 | No diagnostic testing |
| 7[d] | Vanishing twin | 30 | 10 | 10 | Trisomy 12 | Normal CVS |
| 8[e] | Vanishing twin | 41 | 13 | 9 | Trisomy 12 and trisomy 15 | Normal amniocentesis |
| 9[f] | Vanishing twin | 39 | 13 | 10 | dup(21)(q21.1q22.3) | Normal amniocentesis |

yr, years; GA, gestational age; wk, weeks; FF, fetal fraction; cfDNA, cell-free DNA; CVS, chorionic villus sampling. [a]One fetus arrested in week 8 of pregnancy. A repeat cfDNA screen carried out later in the pregnancy was normal. [b]Suspicion of a vanishing twin at 16 weeks of gestation. [c]At 13+5 weeks of gestation, two embryos are present (one fetus had no cardiac activity). [d]Twin gestation at beginning of pregnancy but at week 10+4 one fetus was not visualized. [e]One fetus arrested in week 8 of pregnancy. Structural heart defects were detected in the surviving fetus. [f]Suspicion of a vanishing twin at 13 weeks of gestation.
